# Olipudase alfa enzyme replacement therapy for acid sphingomyelinase deficiency (ASMD): sustained improvements in clinical outcomes after 6.5 years of treatment in adults

**DOI:** 10.1186/s13023-023-02700-x

**Published:** 2023-04-25

**Authors:** Robin H. Lachmann, George A. Diaz, Melissa P. Wasserstein, Nicole M. Armstrong, Abhimanyu Yarramaneni, Yong Kim, Monica Kumar

**Affiliations:** 1 National Hospital for Neurology, University College London Hospitals, London, UK; 2 Icahn School of Medicine at Mount Sinai, New York, NY US; 3 Children’s Hospital at Montefiore, Albert Einstein College of Medicine, Bronx, NY US; 4 Sanofi, Cambridge, MA USA; 5 Sanofi, Bridgewater, NJ USA; 6 Sanofi, Paris, France

**Keywords:** Olipudase alfa, Recombinant human acid sphingomyelinase, Acid sphingomyelinase deficiency, Niemann-Pick disease type B and type A/B

## Abstract

**Background:**

Enzyme replacement therapy with olipudase alfa, a recombinant human acid sphingomyelinase (rhASM), is indicated for non-central nervous system manifestations of acid sphingomyelinase deficiency (ASMD) in children and adults. An ongoing, open-label, long-term study (NCT02004704) assessed the safety and efficacy of olipudase alfa in 5 adults with ASMD. Results: After 6.5 years of treatment, there were no discontinuations, no olipudase-alfa-related serious adverse events, and no new safety signals compared to earlier assessments. Most treatment-emergent adverse events were mild in intensity (1742/1766, 98.6%). Among treatment-related adverse events (n = 657), more than half were considered infusion-associated reactions (n = 403, 61.3%) such as headache, nausea, abdominal pain, arthralgia, pyrexia, and fatigue. No patient developed neutralizing anti-drug antibodies to cellular uptake, and there were no clinically significant adverse changes in vital signs, hematology, or cardiac safety parameters. Improvements (decreases) in spleen and liver volumes progressed through 6.5 years (mean changes from baseline of -59.5% and  -43.7%, respectively). There was a mean increase in diffusing capacity of the lung for carbon monoxide from baseline of 55.3%, accompanied by improvements in interstitial lung disease parameters. Lipid profiles at baseline indicated dyslipidemia. All patients had sustained decreases in pro-atherogenic lipid levels and increases in anti-atherogenic lipid levels following olipudase alfa treatment.

**Conclusions:**

Olipudase alfa is the first disease-specific treatment for ASMD. This study demonstrates that long-term treatment with olipudase alfa is well-tolerated and is associated with sustained improvements in relevant disease clinical measures.

NCT02004704 registered 26 November 2013, https://clinicaltrials.gov/ct2/show/NCT02004704?term=NCT02004704&draw=2&rank=1.

**Supplementary Information:**

The online version contains supplementary material available at 10.1186/s13023-023-02700-x.

## Introduction

Acid sphingomyelinase deficiency (ASMD) is an autosomal recessive lysosomal storage disease resulting from biallelic pathogenic variants in the *SMPD1* gene encoding the lysosomal enzyme acid sphingomyelinase (ASM) [1]. ASMD presents a clinical spectrum of symptom severity and disease burden with sphingomyelin accumulation in multiple organs, causing visceral disease in all cases and neurodegeneration in more severe phenotypes. ASMD type A, with an infantile neurovisceral phenotype (historically known as Niemann-Pick disease type A [NPD A]) uniformly results in death before three years of age [2]. ASMD type B (NPD B) and ASMD type A/B (NPD A/B) are the chronic visceral and chronic neurovisceral phenotypes, respectively, and can present from infancy through to adulthood [3, 4]. Clinical signs include hepatosplenomegaly, liver dysfunction, interstitial lung disease, dyslipidemia, and thrombocytopenia [5–7] in both phenotypes, and neurological symptoms in ASMD type A/B [3]. Growth restriction during childhood and bone disease are common features of ASMD type B and A/B [8]. Pulmonary and liver disease are the leading causes of early mortality among individuals with chronic forms of ASMD [6, 7, 9, 10].

Olipudase alfa (Xenpozyme™), a recombinant human ASM (rhASM), is an intravenous enzyme replacement therapy (ERT) approved for the treatment of non-central nervous system manifestations in children and adults with ASMD in the European Union, the United States, and other countries. Olipudase alfa was effective and well-tolerated in a placebo-controlled trial in adults and an open-label trial in children with chronic ASMD [11–13].

A dose escalation regimen minimizes potential risks associated with the rapid metabolism of accumulated sphingomyelin and the generation of pro-inflammatory products that may induce infusion associated reactions (IARs) or transient liver enzyme elevations [11, 12, 14]. The regimen was first evaluated in a 6-month Phase 1b study in five adults with chronic ASMD who were administered initial doses of 0.1 mg/kg of olipudase alfa followed by stepwise biweekly increases to reach the target dose of 3.0 mg/kg [15]. Dose escalation was successful, and olipudase alfa treatment showed a favorable safety profile and improved clinical endpoints and disease biomarkers [15]. Results were maintained after 30 and 42 months of follow-up [16, 17]. The present analysis shows progressive improvements in multiple clinically relevant disease parameters and a sustained safety profile after 6.5 years of olipudase alfa treatment.

## Methods

### Patients and Study Design

This ongoing, open-label, long-term study (NCT02004704; EudraCT Number: 2013-000051-40) follows the five adults with chronic ASMD enrolled in the United States and the United Kingdom who previously completed the Phase 1b study [15]. Data were analyzed after 6.5 years of treatment (data cutoff 01 March 2021). The Institutional Review Board or Ethics Committee at each site approved the protocol, and all patients provided written informed consent. The study was conducted according to Good Clinical Practice and in accordance with the principles of the Declaration of Helsinki.

Eligibility criteria for the Phase 1b study have been described [15]. Key clinical criteria included diffusing capacity for carbon monoxide (DL_CO_) between 20% and 80% of predicted normal value, spleen volume ≥ 6 multiples of normal (MN), and platelet count ≥ 60 × 10^9^/L. Patients continued in the long-term study at the same olipudase alfa dose they were receiving at the end of the Phase 1b study.

Olipudase alfa is produced in a Chinese hamster ovary cell line using recombinant DNA technology. The manufacturing process evolved during olipudase alfa clinical development. All five original participants in the Phase 1b study who are now being followed in the long-term study received product manufactured by an early process for the first four years of treatment and were then switched to product manufactured using an updated process (which differed primarily by the production scale) that is equivalent to the commercial olipudase alfa product.

### Outcome measures and analyses

Safety assessments included standard hematologic and chemistry panels and continuous adverse event monitoring, including infusion-associated reactions (IARs), as previously described [15]. Disease biomarkers included chitotriosidase activity and plasma lyso-sphingomyelin level. The presence of anti-drug antibodies was assessed as previously described [15].

Spleen and liver volumes were determined from abdominal MRI and expressed as multiples of normal (MN), where normal spleen volume was assumed to be 0.2% of body weight and normal liver volume to be 2.5% of body weight [18]. Severe and moderate organomegaly were defined as > 15 and > 5 to ≤ 15MN, respectively, for splenomegaly and > 2.5 and > 1.25 to ≤ 2.5MN, respectively, for hepatomegaly [18]. Percent predicted DL_CO_ adjusted for hemoglobin was calculated using standardized formulas [19, 20], and the severity of impairment was categorized as > 80% normal/no impairment; >60% to ≤ 80% mild; >40% to ≤ 60% moderate; <40% severe [21]. High-resolution computed tomography (HRCT) imaging of the lungs was used to assess interstitial lung disease. HRCT images were scored subjectively for ground glass appearance, interstitial lung disease, and reticulonodular density on a scale from 0 (no disease) to 3 (severe disease) as previously described [15]. Spirometry for volumetric pulmonary function tests (forced vital capacity, forced expiratory volume in one second, and total lung capacity) was performed using American Thoracic Society guidelines [22].

Fasting plasma lipid measurements included profiles of pro-atherogenic [total cholesterol (TC), low-density lipoprotein (LDL-C), triglycerides)] and anti-atherogenic [high-density lipoprotein (HDL-C)] lipids to assess dyslipidemia over time. Platelet counts were assessed using a thrombocytopenia threshold of 150 × 10^9^/L.

Bone mineral density (BMD) was determined from dual-energy X-ray absorptiometry (DXA) bone scan images of the lumbar spine and both femurs, and T- and Z-scores were determined [23] and assessed using the International Society for Clinical Densitometry guidance [24].

Patient-reported outcomes (PROs) included the validated Brief Fatigue Inventory (BFI) [25] and Brief Pain Inventory-Short Form (BPI-SF) [26] questionnaires [using 11-point scales from 0 (absence) to 10 (worst) ]. Patients rated fatigue from 0 (no fatigue) to 10 (worst fatigue) and scores were categorized as mild (1–3), moderate (4–6), and severe (7–10). Patients rated pain intensity from 0 (no pain) to 10 (worst pain), which was categorized as mild (1–4), moderate (5–6), and severe (7–10) [26].

## Statistical methods

Descriptive statistics were provided for categorical and continuous variables. The change or percentage change from baseline was analyzed using the analysis of covariance (ANCOVA) method adjusting for baseline value, and least square means and standard errors were determined. No multiplicity adjustment was conducted, and all p-values based on the ANCOVA model are nominal.

## Results

### Patients and exposure

Baseline characteristics have been previously described [15, 16] and are listed in Supplemental Table S1. In summary, baseline age ranged from 22 to 47. Participants (3 male, 2 female) had moderate to severe splenomegaly, mild to moderate hepatomegaly, mild to moderately impaired DL_CO_, and a pro-atherogenic lipid profile.

Four of the five individuals remained at the 3 mg/kg olipudase alfa target dose through the 6.5 years of treatment. One individual had a dose reduction to 1 mg/kg at week 74 (1.4 years) due to adverse events as previously described [15], resumed the 3 mg/kg dose at week 246 (4.7 years), and has remained at the target dose through year 6.5.

### Safety

There were no deaths, olipudase alfa-related serious adverse events, or permanent discontinuations during olipudase alfa treatment. The overview of adverse events through year 6.5 is shown in Table 1. All individuals had at least one adverse event; almost all events (1742/1766, 98.6%) were mild in intensity. One individual had five serious events unrelated to treatment (increased endometrial thickness, acid reflux, ovarian cyst, uterine fibroid, and squamous cell skin carcinoma). Four of five participants had at least one treatment-related adverse event, over half of which (403/657, 61.3%) were considered IARs (as algorithm defined-see Table 1). Among events considered to be IARs by investigators (protocol-defined IARs, n = 116), the most common (≥ 5% of events) included headache, nausea, abdominal pain, arthralgia, pyrexia, and fatigue (Table 1). The frequency of protocol-defined IARs over time is shown in Additional File 1, Supplemental Figure S1. The majority 91/116 (78%), occurred within the first 6months of treatment. Two individuals had three events (flushing and two events of urticaria) that were considered hypersensitivity-related IARs, and that occurred within the first 4 years of treatment. These were managed with supportive care, and none required a change in dose. No new safety concerns have emerged since the last assessments [16, 17].

**Table 1:** Summary of Adverse Events

Profile of Treatment-Emergent Adverse Events	Patients n (%)	Events n	
**Any Event**	5 (100)	1766	
Mild	5 (100)	1742	
Moderate	2 (40)	23	
Severe	1 (20)	1	
**Serious Events**	1 (20)	5	
**Serious Events Related to Treatment**	0	0	
**Deaths, Permanent Discontinuations**	0	0	
Protocol-defined IAR*	4 (80)	116	
Algorithm-defined IAR**	5 (100)	403	
**Treatment-Related Events**	4 (80)	657	
**Most Common Treatment-Related Events** **(≥ 5% of all related events)**	Patients n (%)	**Events** **n**	IAR* **n (%)**
Headache	3 (60)	75	24 (32)
Nausea	4 (80)	71	18 (25)
Abdominal pain	4 (80)	56	9 (16)
Arthralgia	4 (80)	50	13 (26)
Pyrexia	3 (60)	46	5 (11)
Fatigue	3 (60)	36	2 (6)

IAR = infusion associated reaction

* Identified as an IAR by the investigator

**All adverse events occurring between the start of infusion and the end of infusion plus 24 h, irrespective of the perceived relation with study treatment

Three individuals developed IgG antibodies to olipudase alfa, two with low responses and one with a persistent ADA response that reverted to ADA negative at the final assessment. None of these individuals tested positive for neutralizing antibodies that interfered with olipudase alfa uptake into cells. Two individuals tested transiently or intermittently positive for inhibition of enzyme catalytic activity, but the relevance of this is unknown given that all clinical parameters continue to improve.

### Efficacy

#### Spleen and liver volumes

**Fig. 1:**
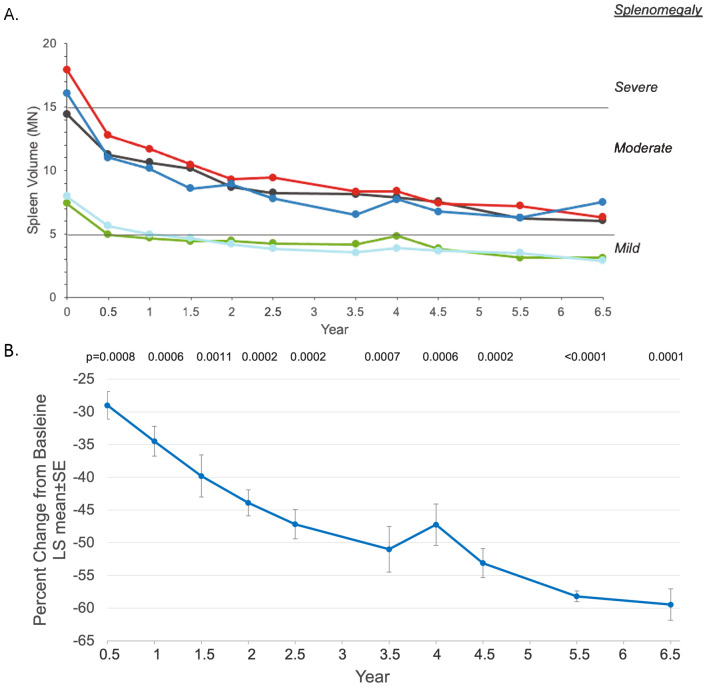
**Assessment of Olipudase Alfa on Spleen Volumes** A. Individual patient spleen volumes by years of olipudase alfa treatment. Spleen volumes were calculated by integrating cross-sectional magnetic resonance images and expressed as multiples of normal (MN) where normal spleen volume was assumed to be 0.2% of body weight [18]. Severe and moderate splenomegaly were defined as > 15 and > 5 to ≤ 15MN, respectively [18]. Cutoffs of MN for severity of splenomegaly are indicated by horizontal lines B. Least square (LS) mean percent changes ± SE of the mean in spleen volume from baseline over time. All p values are nominal

**Fig. 2:**
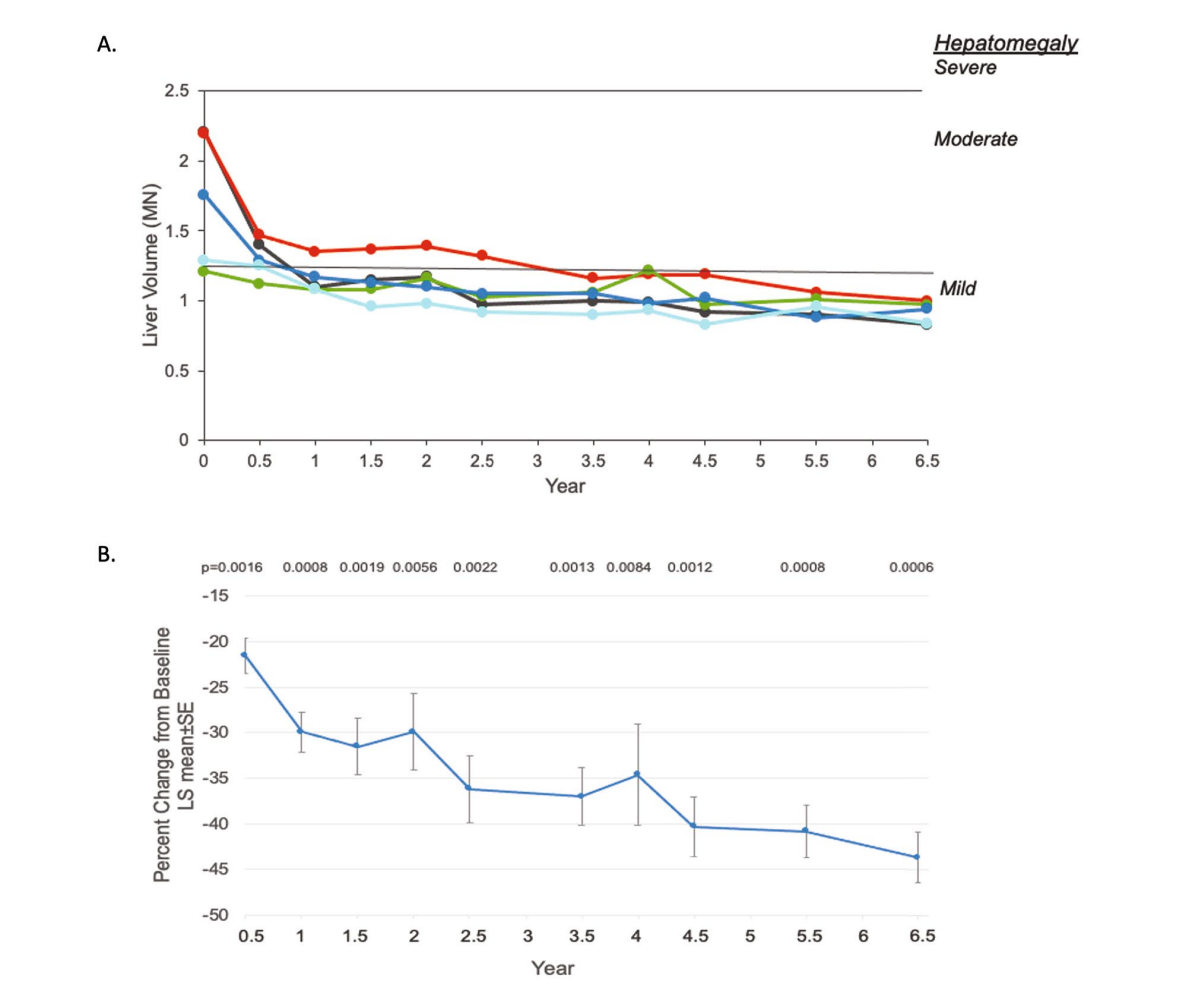
**Assessment of Olipudase Alfa on Liver Volumes** A. Individual patient liver volumes by years of olipudase alfa treatment. Liver volumes were calculated by integrating cross-sectional magnetic resonance images and expressed as multiples of normal (MN) where normal liver volume was assumed to be 2.5% of body weight [18]. Severe and moderate hepatomegaly were defined as > 2.5 and > 1.25 to ≤ 2.5MN [18], respectively. Cutoffs of MN for severity of hepatomegaly are indicated by horizontal lines B. Least square (LS) mean percent changes ± SE of the mean in liver volume from baseline over time. All p values are nominal

Clinically significant decreases in spleen and liver volumes were reported at 6 months [15] and 30 months [16], and these parameters continued to improve through year 6.5 (Figs. 1 and 2).

Splenomegaly improved in all five participants (Fig. 1A). Please note that there is consistent use of colors across graphs for all individual data in the main text and supplemental material. Two individuals with severe splenomegaly at baseline improved to moderate splenomegaly, and two out of three patients with moderate splenomegaly at baseline improved to mild splenomegaly. Mean ± SD spleen volumes decreased from 12.8 ± 4.8 MN at baseline to 5.2 ± 2.1 at year 6.5 (LS mean decrease from baseline ± SE was 7.6 ± 0.4, P = 0.0003; LS mean percent decrease ± SE from baseline was 59.5 ± 2.4, P = 0.0001). The LS mean percent change from baseline decreased (improved) throughout treatment (Fig. 1B).

Hepatomegaly improved for all participants and was mild or absent by year 6.5 (Fig. 2A). Mean liver volumes decreased from 1.7 MN at baseline to 0.92 MN (LS mean decrease from baseline ± SE was 0.82 ± 0.04, P = 0.0003; LS mean percent decrease ± SE from baseline was 43.7 ± 2.8%, P = 0.0006). As with spleen volume, the LS mean percent change in liver volume from baseline decreased (improved) throughout treatment (Fig. 2B).

#### Lung disease

**Fig. 3:**
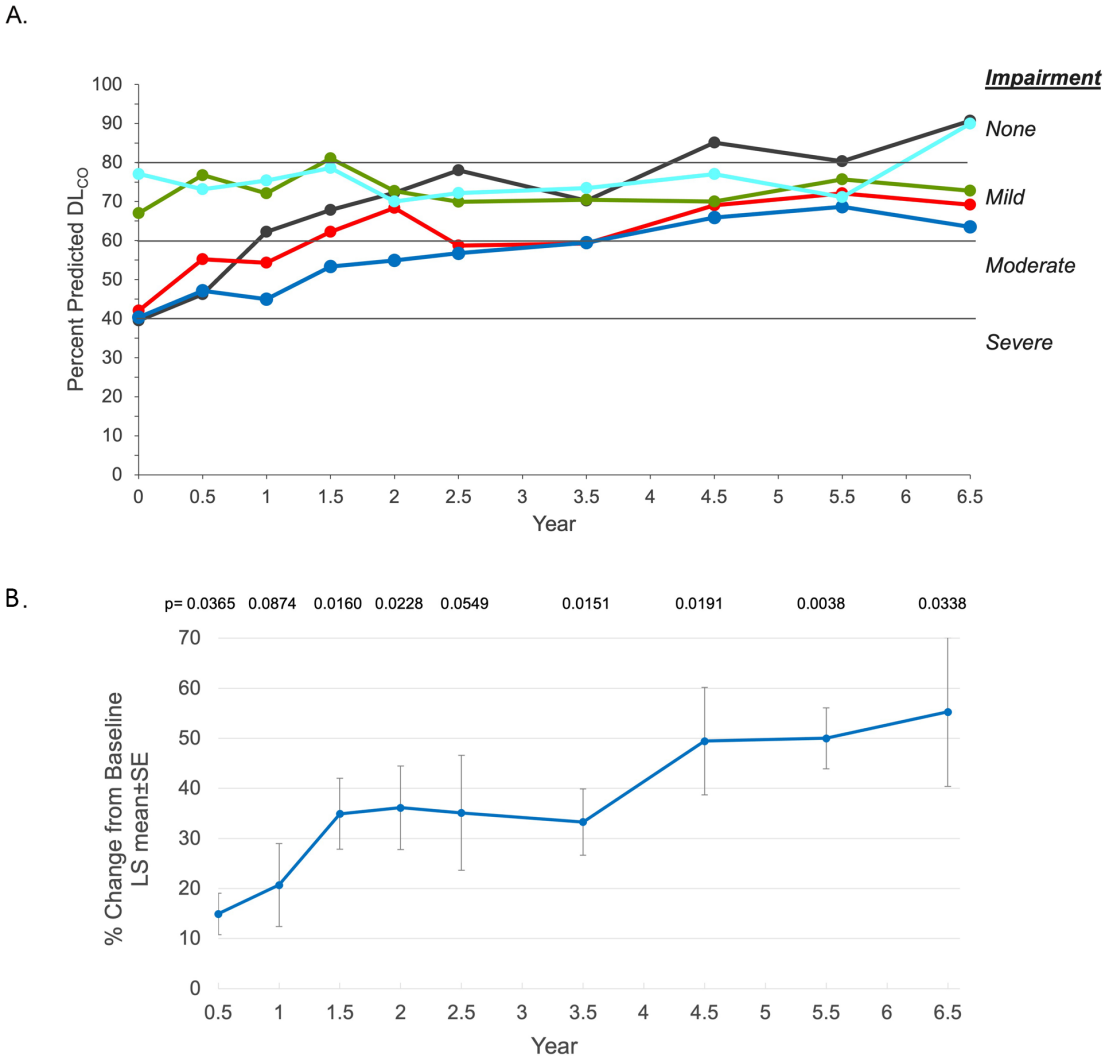
**Diffusing capacity of the lung for carbon monoxide (DL**_**CO**_ **) adjusted for hemoglobin (Hb)** A. Individual patient percent predicted DL_CO_ values. Cutoffs for impairment are indicated by horizontal lines for individual patient data (> 80% normal/no impairment, > 60% to ≤ 80% mild impairment, 40–60% moderate impairment, and < 40% severe impairment) [21] B. Least square (LS) mean percent change from baseline ± SE over time. All p values are nominal

Diffusing capacity of the lung as assessed by the percent predicted DL_CO_ adjusted for hemoglobin improved numerically in all individuals relative to baseline (Fig. 3A). Three individuals with borderline severe impairment at baseline improved to mild (n = 2) or no impairment (n = 1) at year 6.5. Mean ± SD % predicted DL_CO_ improved from 53.2 ± 17.6 at baseline to 77.2 ± 12.4 at year 6.5. LS mean ± SE change from baseline was 24.0 ± 6.0 (P = 0.0274), with a LS mean ± SE percent change from baseline of 55.3 ± 14.9% (P = 0.0338) at year 6.5. Figure 3B shows the improvement % predicted DL_CO_ LS mean percent change from baseline over time.

**Fig. 4:**
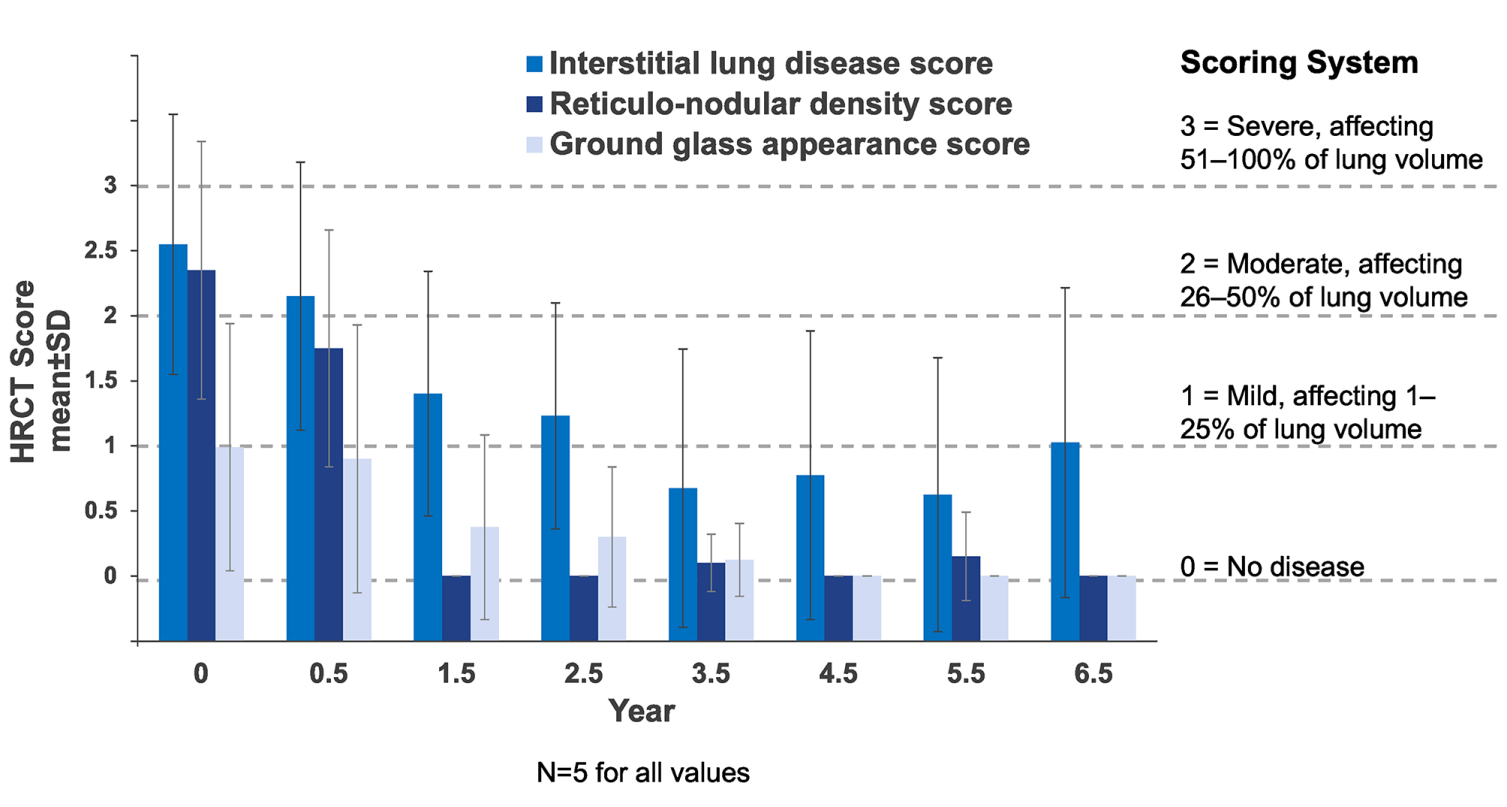
**Assessment of interstitial lung disease by high-resolution computed tomography (HRCT)**. Mean HRCT scores over time for ground glass appearance, interstitial lung disease, and reticulo-nodular density. Scoring is based on a 4-point system where 0 = No disease; 1 = Mild (affecting 1–25% of the lung volume); 2 = Moderate (affecting 26–50% of the lung volume); 3 = Severe (affecting 51–100% of the lung volume)

**Fig. 5:**
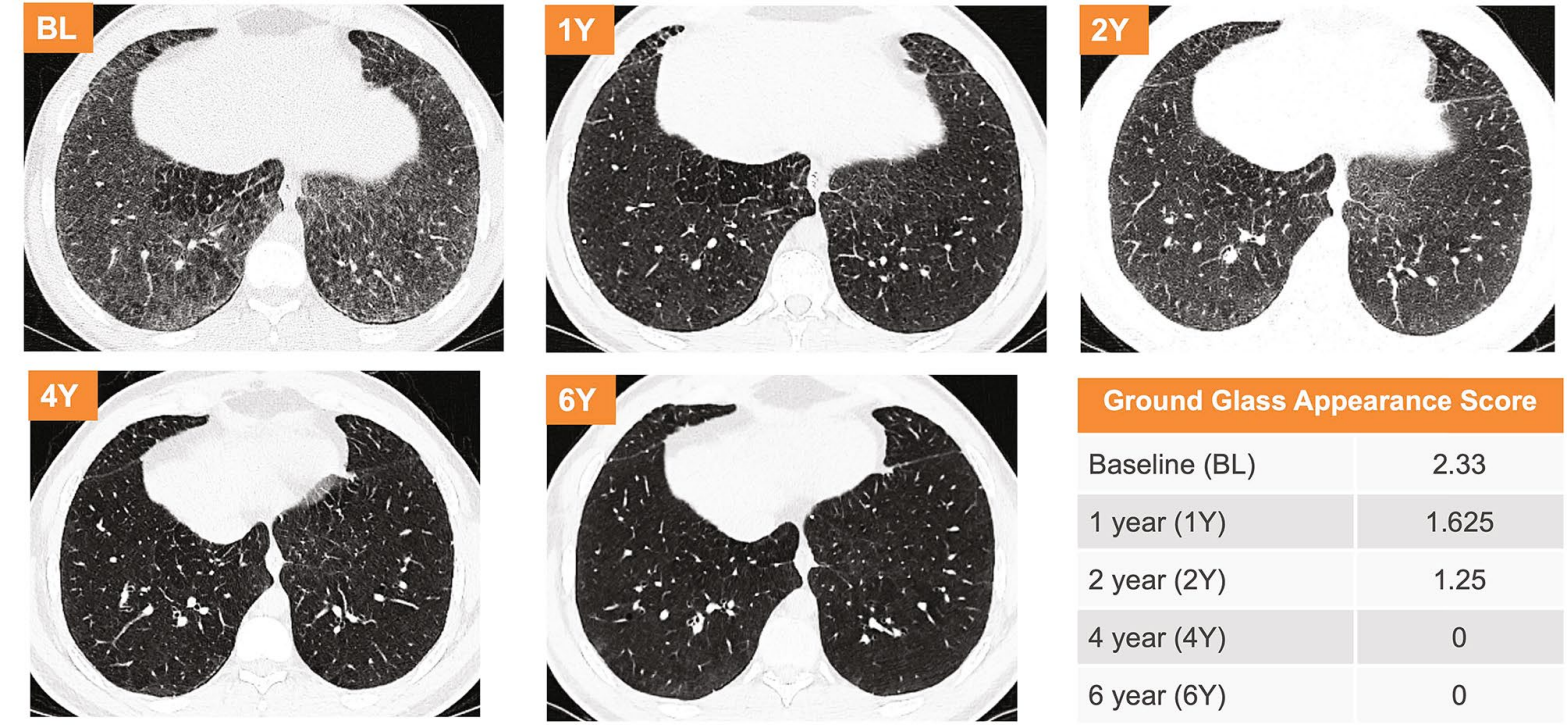
**Ground Glass Appearance HRCT Images and Scores.** Images and scores at baseline and through year 6 for the individual with the highest (worst) baseline score

Interstitial lung disease was assessed using lung imaging to detect characteristic parenchymal HRCT patterns, including ground glass appearance, interstitial disease, and reticulonodular density. Mean scores based on the percent of lung volume affected for each component are shown in Fig. 4. Mean scores of all components improved and completely resolved for ground glass appearance and reticulonodular density by year 6.5. There were both inter- and intra-individual variability in ILD scores over time. Individual data over time for interstitial lung disease and ground glass appearance scores are shown in Additional File 1, Supplemental Figure S2A and B. HRCT images for ground glass appearance over time for the individual with the worst baseline scores are shown in Fig. 5. Their ground glass appearance score of 2.33 at baseline was in the moderate category (affecting 26–50% of the lung volume) and resolved (score of 0) by year 4.

Spirometry values for forced vital capacity, forced expiratory volume in 1 s, and total lung capacity were within normal ranges (percent predicted ≥ 80%) at baseline [15] and throughout the study (Additional File 1, Supplemental Figure S3).Three individuals with the lowest values at baseline (between 78% and 95%) had improvements in all three measures at year 6.5 (between 94% and 112%) (Additional File 1, Supplemental Figure S3). The dark blue lines in the lung spirometry graphs represent the individual whose HRCT images are shown in Fig. 5. The individual had a % predicted baseline value of 90, 78.6, and 92.5 for FVC, FEV1, and TLC, respectively. All three parameters were improved at 6.5 years relative to baseline (% change from baseline in % predicted value of 23%, 20%, and 18%, respectively).

#### Plasma lipid profiles

**Fig. 6:**
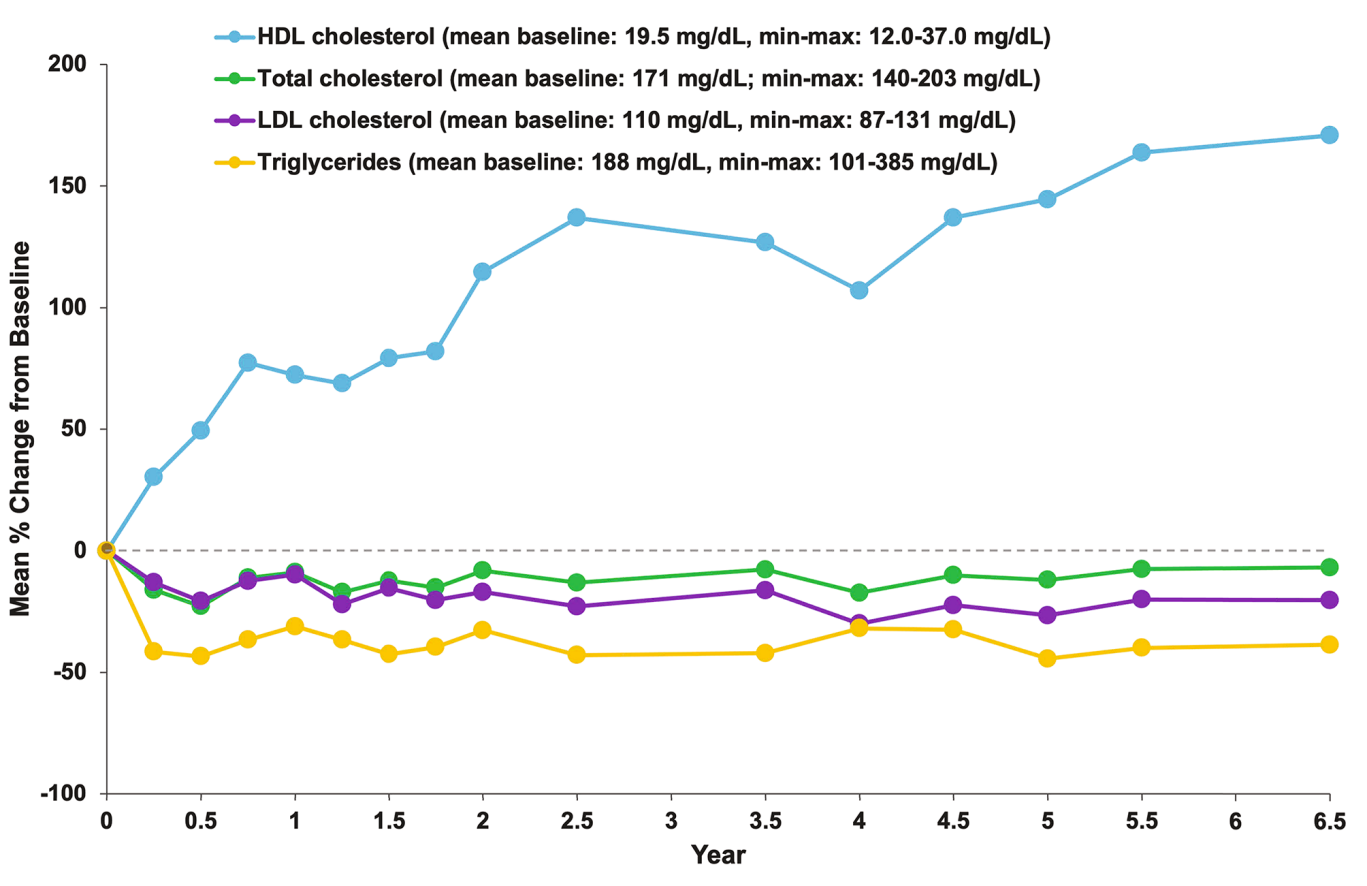
**Plasma Lipid Profiles Over Time.** Mean baseline values and mean percent changes from baseline are shown for pro-atherogenic (total cholesterol, LDL cholesterol and triglycerides), and anti-atherogenic l (HDL cholesterol) lipids. Data are shown for timepoints at which all five patients had a complete lipid profile

Baseline values and the mean percent changes from baseline in plasma lipids are shown in Fig. 6. Progressive reductions from baseline in pro-atherogenic lipid profiles (total cholesterol, LDL-cholesterol, and triglycerides) observed at month 6 [15] and year 3.5 [17] remained stable through year 6.5. In contrast, levels of the anti-atherogenic marker HDL-C continued to increase (improve) throughout olipudase alfa treatment.

#### Bone mineral density

**Fig. 7:**
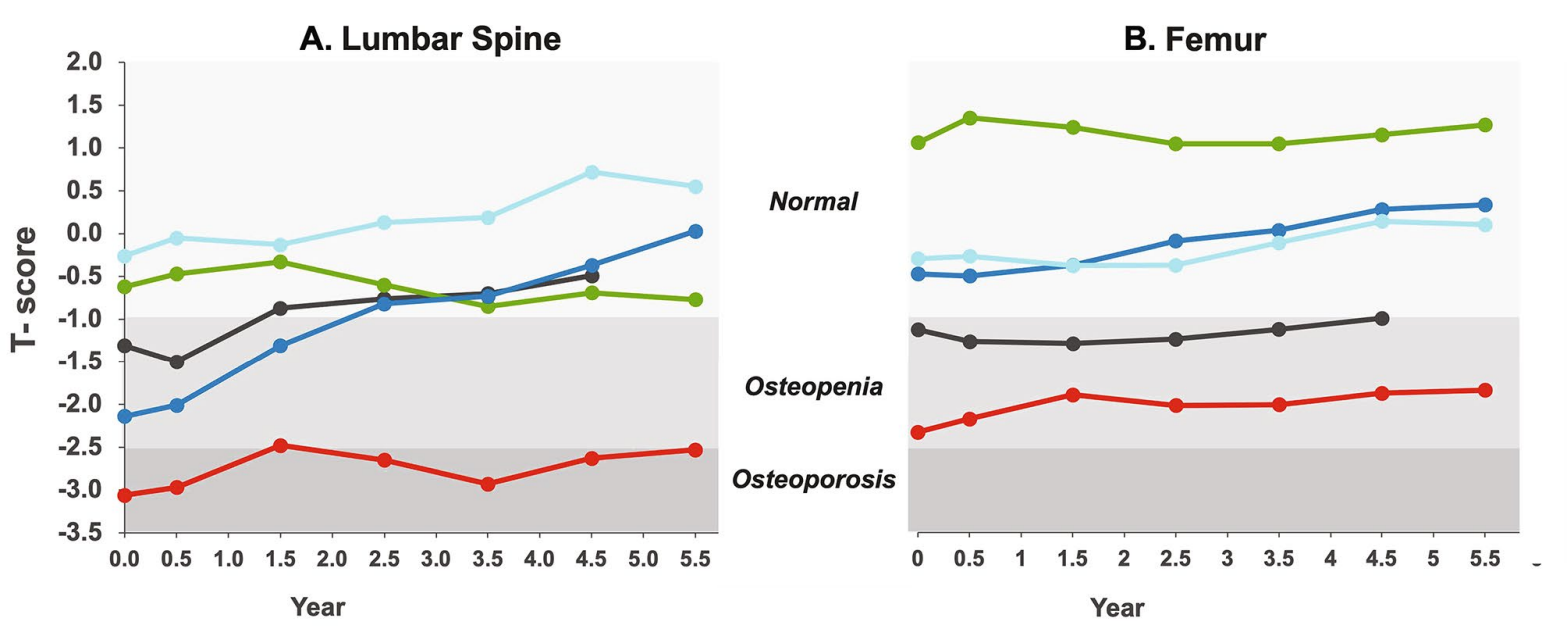
**Assessment of Bone Mineral Density Over Time.** Individual lumbar spinal and femur T-scores

At the data cutoff, bone mineral density data at year 5.5 were available. At baseline, the mean ± SD spinal T-score was − 1.5 ± 1.1 (osteoporosis indicated by T-score below − 2.5; osteopenia ranges between − 2.5 and − 1) and the mean Z-score (-1.4 ± 1.3) indicated normal BMD (-2.0 low BMD cutoff). Mean spinal T- and Z-scores improved at year 5.5 (-0.7 ± 1.4 and − 0.4 ± 1.1, respectively). Individual BMD spinal T-scores are shown in Fig. 7A. Two of five individuals with T-scores in the osteopenia range improved to normal values. A 32-year-old individual with a baseline spinal T-score of -3.1 had a value of -2.5 at 5.5 years. Results for individual spinal Z-scores over time were similar to those for T-scores (data not shown). Mean femur T- and Z-scores were in the normal ranges at baseline (-0.4 ± 1.4 and − 0.3 ± 1.5, respectively) and were increased at year 5.5 (0.3 ± 1.4 and 0.5 ± 1.6, respectively) relative to baseline. Individual femur T-scores at baseline were within normal (n = 3) or osteopenia (n = 2) ranges and increased slightly over time (Fig. 7B).

#### Other endpoints

At baseline, mean ± SD platelet count was 118 ± 35 × 10^9^/L [15] and thrombocytopenia (platelet count below 150 × 10^9^/L) was observed in four of five individuals. At year 6.5, the mean ± SD improved to 161 ± 41 × 10^9^/L, and platelet counts remained below normal for two individuals. The LS mean percent change from baseline ± SE at year 6.5 was 38.5 ± 6.4%, P = 0.0093.

Improvements in liver enzymes observed at 6 months [15] were stable at year 3.5 [17] and remained stable through year 6.5 (data not shown).

#### Biochemical disease marker Assessment

Mean ± SD plasma lyso-sphingomyelin level at baseline was significantly elevated (398.6 ± 226.8 µg/L; upper limit of normal ULN = 10 µg/L) and decreased by 6 months to levels that remained stable through year 6.5 (year 6.5 mean ± SD 58.4 ± 13.8; Additional File 1, Supplemental Figure S4A). The LS mean ± SE percent change from baseline at year 6.5 was − 80.5 ± 1.9%, p < 0.0001.

Pre-infusion serum chitotriosidase activity, a measure of macrophage activation, steadily decreased from a mean ± SD of 1016 ± 479 µmol/L/h at baseline and remained below the upper limit of normal for chitotriosidase (≤ 181 µmol/L/h) from year 3 through year 6.5 (year 6.5 mean ± SD 66 ± 42 µmol/L/h; Additional File 1, Supplemental Figure S4B). The LS mean ± SE percent change from baseline at year 6.5 was − 92.9 ± 1.7% (p < 0.0001). Data were adjusted to account for two patients heterozygous for a common 24-base pair duplication that reduces serum chitotriosidase activity (values multiplied by 2).

#### Patient-reported outcomes

The mean ± SD baseline BFI score (fatigue at its usual in the last 24 h) was moderate, 5.4 ± 2.2, and decreased to 2.4 ± 1.5 at 6.5 years (mild). BPI average pain score at baseline was 3.2 ± 2.8 (mild) and decreased to 2.4 ± 2.3 at 6.5 years. Worst pain severity scores decreased from moderate at baseline (4.6 ± 3.1) to mild (3.8 ± 3.6) at 6.5 years.

## Discussion

Olipudase alfa is the first disease-modifying treatment approved for adults and children with ASMD. Approval was based on the outcomes of a 1-year placebo-controlled trial in 36 adults with ASMD [11] and a 1-year open-label study in 20 children with chronic ASMD [12]. This smaller open-label study of five adults with ASMD treated with bi-weekly infusions of olipudase alfa has provided long-term data on safety and efficacy [15–17]. We now report sustained improvements in clinical measures of disease over 6.5 years of treatment. The biochemical markers of disease burden lyso-sphingomyelin and chitotriosidase were elevated at baseline, declined in the first 1–3 years of treatment [15, 16], and remained within, or close to, normal levels through 6.5 years. The improvements in multiple clinical endpoints reported after 2.5 and 3.5 years of olipudase alfa treatment [16, 17] remained stable or were further improved after 6.5 years of treatment and reflected normalization or near normal values. The results of this study are consistent with reported safety and efficacy in the two larger trials in adults and children [11–13].

Progressive liver disease, cirrhosis, and liver failure contribute to morbidity and early mortality in adults with chronic ASMD [9]. As reported in previous publications, these five adults showed significant and sustained improvements in liver sphingomyelin burden within the first year of olipudase alfa treatment [15], and the clearance of excess sphingomyelin in hepatocytes and Kupfer cells through year 3.5 of treatment resulted in improved biochemical parameters of liver function [17]. The severity of hepatomegaly steadily decreased over 6.5 years of treatment in all participants. Dyslipidemia, which typically worsens over time in adults with chronic ASMD [4, 7], improved over the 6.5 years of treatment with a lipid profile showing a continued increase in anti-atherogenic lipid.

Splenomegaly is a prominent clinical manifestation of ASMD and contributor to disease burden [27]. Among 17 adults with mild to very severe ASMD, abdominal pain/discomfort and abdominal distension were prominent symptoms that impacted multiple aspects of their daily lives [28]. Olipudase alfa improved splenomegaly in all study participants and was accompanied by increases in platelet counts, indicating the correction of secondary hypersplenism contributing to thrombocytopenia [29].

The presentation of pulmonary symptoms in chronic ASMD is varied, but ASMD type B and A/B result in worsening interstitial lung disease with age [4]. However, patients may not have overt symptoms or abnormal lung function tests [30, 31], although exercise intolerance can develop over time. In a prospective 11-year natural history study of 59 patients, individuals with severe or moderate ILD tended to be younger than those with absent or mild disease [7]. In some cases, pulmonary disease has been the clinical manifestation that drives diagnosis in adults. Progressive dyspnea (with ILD on imaging) prompted a clinical workup and resulting diagnosis of ASMD in two adult siblings age 52 and 61 years, [32]. Pulmonary morbidities in chronic ASMD, including respiratory infections and respiratory failure, contribute to early mortality [7, 9]. In a complicated disease such as ASMD where radiologic findings of ILD may not show strong correlation with the results of pulmonary function tests [30], it is important to use multiple measures to assess pulmonary outcomes with treatment. Olipudase alfa treatment significantly improved lung diffusing capacity in all five individuals, resulting in no or mild impairment after 6.5 years, with improvements in spirometry in some cases. In parallel, radiographic imaging of lung parenchymal features over time indicated resolution of ground glass appearance and improvement in ILD scores. It is important to note that there are two components of ILD in ASMD: alveolar infiltration and infiltration of the intra-alveolar septum (as is seen in other interstitial lung diseases such as pulmonary fibrosis). Both contribute to reducing DL_CO_, and alveolar infiltrates can also reduce alveolar volume. On HRCT, ground glass appearance assesses alveolar infiltration while ILD assesses septal disease. Alveolar infiltrates responded dramatically and quickly to olipudase alfa (ground glass appearance resolves) but it appears that, in some individuals, septal disease may be slower to respond, perhaps due to fibrosis.

Skeletal complications are also prominent features of chronic ASMD. Assessment of bone mineral density in 46 individuals with ASMD type B compared to unaffected healthy children and adults found that most adults with ASMD have osteopenia or osteoporosis, with the degree of splenomegaly correlating inversely with lumbar spine bone mineral density Z-scores [33]. Skeletal fractures are reported for 53% of adults and 25% of children [33]. In the present analysis, the individual with the largest spleen had the lowest lumbar spine and femur T-scores. T-scores improved modestly over time; however, the small sample size precludes any definitive conclusions on the impact of olipudase alfa on skeletal outcomes.

No new safety issues emerged with long-term olipudase alfa treatment. The safety profile through year 6.5 was similar to that reported for the original Phase 1b 6-month study [15] and after 30 months of treatment [16]. Importantly, no individual has discontinued treatment during the study. Inherent limitations of this open-label long-term study include the small number of study participants. However, the improvements in all individuals in the clinical manifestations of ASMD highlight the ability to assess treatment benefits for rare diseases with small patient numbers.

### Conclusions

Treatment with olipudase alfa for 6.5 years is well-tolerated and is associated with sustained improvements in relevant disease clinical measures. Organomegaly, pulmonary function, and hematological and biochemical parameters remain within, or continue to trend toward, normal ranges.

## Electronic supplementary material

Below is the link to the electronic supplementary material.

Supplementary Material 1
Additional file 1: Supplemental Table S1. Demographics and Baseline Characteristics.
Additional file 1: Supplemental Figure S1. Lung spirometry values over time. Individual responses for derived percent predicted forced vital capacity (FVC) (A), forced expiratory volume in 1 s (FEV1) (B), and total lung capacity (TLC) (C).
Additional file 1: Supplemental Figure S2 Mean plasma lyso-sphingomyelin levels (A) and mean serum chitotriosidase activity (B) over Time.

## Data Availability

Qualified researchers may request access to patient level data and related study documents including the clinical study report, study protocol with any amendments, blank case report form, statistical analysis plan, and dataset specifications. Patient level data will be anonymized, and study documents redacted to protect the privacy of trial participants. Further details on Sanofi’s data sharing criteria, eligible studies, and process for requesting access can be found at: https://vivli.org.
