# Supplementary material for: Olipudase alfa enzyme replacement therapy for acid sphingomyelinase deficiency (ASMD): sustained improvements in clinical outcomes after 6.5 years of treatment in adults
Source: Orphanet J Rare Dis. 2023 Apr 25;18:94. doi: 10.1186/s13023-023-02700-x (PMC10131350; doi:10.1186/s13023-023-02700-x)

**Additional File 1**

**Supplemental Table S1: Demographics and Baseline Characteristics [15]**

|  | **Study Participant*** | | | | |  |
| --- | --- | --- | --- | --- | --- | --- |
|  | **1** | **2** | **3** | **4** | **5** |  |
|  | Male | Female | Female | Male | Male | **Mean (SD)** |
| **ASMD Symptom onset age (years)** | 2 | 1 | 6 | 0 | 12 | 4.2 (4.9) |
| **ASMD Diagnosis age (years)** | 2 | 2 | 12 | 8 | 12 | 7.2 (5.0) |
| **Age at first olipudase alfa infusion (years)** | 31 | 32 | 47 | 28 | 22 | 32.6 (9.4) |
| **Spleen Volume (MN)^a^** | 14.49 | 17.92 | 7.41 | 16.07 | 7.96 | 12.77 (4.81) |
| **Liver Volume (MN)^a^** | 2.23 | 2.20 | 1.21 | 1.76 | 1.29 | 1.74 (0.48) |
| **DL_CO_ (% predicted)^b^** | 43.7 | 48.0 | 77.0 | 43.0 | 80.0 | 58.3(18.5) |
| **Total cholesterol: high-density lipoprotein ratio^c^** | 14.7 | 10.6 | 5.5 | 15.0 | 6.4 | 10.4 (4.5) |

ASMD = acid sphingomyelinase deficiency; DL_CO_=lung diffusion of carbon monoxide

*Note that colors match the colors used in line graphs of individual data

^a^ MN, multiples of normal calculated assuming normal spleen volume (L) is 0.2% body weight (kg), and normal liver volume (L) is 2.5% body weight (kg)

^b^ Normal DLco >80%; Mildly reduced >60% to ≤ 80%; Moderately reduced 40-60%; Severely reduced < 40%

c Ratios >5 may indicate higher atherogenic risk

**Supplemental Figure S1. Infusion associated reactions (IARs).** The number of IARs over time is indicated for 6-month intervals of olipudase alfa treatment. The proportion of individuals with at least 1 IAR during the time interval is shown below the graph.

**
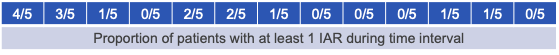
**

**Supplemental Figure S2. Assessment of interstitial lung disease by high-resolution computed tomography (HRCT). Individual HRCT scores over time.**

A. Interstitial lung disease (ILD) scores.

B. Ground glass appearance (GGA) scores.

Scoring is based on a 4-point system where 0 = No disease; 1 = Mild (affecting 1-25% of the lung volume); 2 = Moderate (affecting 26-50% of the lung volume); 3 = Severe (affecting 51-100% of the lung volume).

**A.**

**B.**

**Supplemental Figure S3. Individudal responses for derived percent predicted values for ung spirometry values over time.**

A. Forced vital capacity (FVC).

B. Forced expiratory volume in 1 second (FEV1).

C. Total lung capacity (TLC).

**A.**

**B.**

**C.**

**Supplemental Figure S4. Mean ± SD pre-infusion plasma lyso-sphingomyelin levels (A) and mean serum chitotriosidase activity (B) over time.** The upper limit of normal for lyso-sphingomyelin in plasma was 10µg/L, and normal chitotriosidase serum levels were ≤ 181 nmol/hr/mL (indicated by horizontal dotted lines). Chitotriosidase values for two individuals heterozygous for the null *CHIT* mutation known to result in inactive enzyme were doubled.

A.


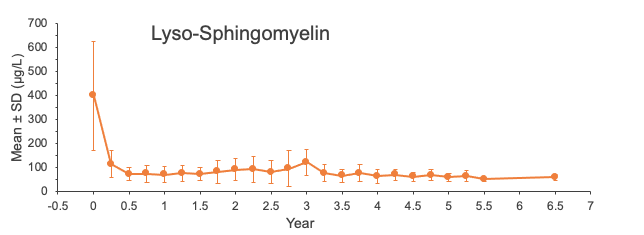


B.


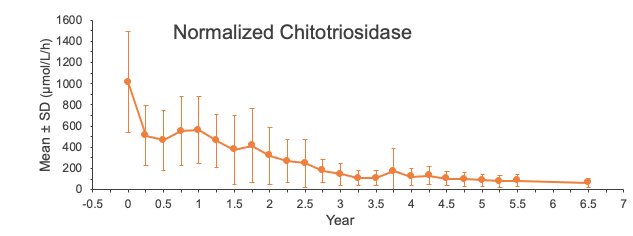

Supplement: Supplementary file 1 — Supplementary Material 1 Additional file 1: Supplemental Table S1. Demographics and Baseline Characteristics. Additional file 1: Supplemental Figure S1. Lung spirometry values over time. Individual responses for derived percent predicted forced vital capacity (FVC) (A), forced expiratory volume in 1 s (FEV1) (B), and total lung capacity (TLC) (C). Additional file 1: Supplemental Figure S2 Mean plasma lyso-sphingomyelin levels (A) and mean serum chitotriosidase activity (B) over Time. [file 13023_2023_2700_MOESM1_ESM.docx]
